# Clinical value at baseline and follow-up of myeloperoxidase-antibodies in ANCA-associated vasculitis

**DOI:** 10.3389/fimmu.2025.1649708

**Published:** 2025-09-01

**Authors:** Gabriel Bossan, Florence Roufosse, Frederic Vandergheynst, Julie Smet, Elie Cogan, Benoit Vokaer

**Affiliations:** ^1^ Department of Internal Medicine, HUB (Hôpital Erasme), Université libre de Bruxelles, Brussels, Belgium; ^2^ Immunology Department, Laboratoire Hospitalier Universitaire de Bruxelles, Université libre de Bruxelles, Brussels, Belgium; ^3^ Department of Internal Medicine, Hôpital Delta (CHIREC), Brussels, Belgium

**Keywords:** ANCA-associated vasculitis, EGPA, GPA, MPA, MPO-ANCA, relapse, predictive value, treatment adjustment

## Abstract

**Background:**

ANCA-associated vasculitides (AAV) are potentially organ- or life-threatening disorders that can cause irreversible damage if treatment is not started in time. The course of the disease may vary once remission has been achieved, with some patients experiencing relapses while others remain in sustained remission. The predictive value of PR3-ANCA for clinical deterioration is well established. However, limited data regarding MPO-ANCA, which has primarily been studied in microscopic polyangiitis (MPA) and eosinophilic granulomatosis with polyangiitis (EGPA), is less clear. This study aims to further clarify the role of MPO-ANCA in predicting relapse.

**Methods:**

We conducted a retrospective review of the medical records of patients for whom positive MPO-ANCA serology was reported by our university laboratory between 2014 and 2024. We included patients who fulfilled the classification criteria for AAV and experienced remission. Remission was defined as a BVAS score of 0 and a prednisone-equivalent dose of less than 7.5 mg/day. We analyzed the impact of MPO-ANCA status (at diagnosis and over time) on the occurrence of relapse separately in patients with EGPA and patients with GPA/MPA. Relapse was defined as a BVAS score of 1 or higher.

**Results:**

A total of 73 patients were included in the study, comprising 22 with EGPA and 51 with GPA/MPA. During follow-up (median 7 and 10yr, respectively), 10 EGPA and 19 GPA/MPA patients experienced a relapse. Baseline MPO-ANCA levels and eosinophil counts at diagnosis were not associated with the risk of relapse. However, an increase in MPO-ANCA levels during follow-up was significantly associated with clinical deterioration in both disease subgroups (positive predictive value 83% in EGPA, 79% in GPA/MPA; *p = 0.0001*). Median interval between an increase in MPO-ANCA levels and relapse was 3.6 ± 2.1 months and 4.6 ± 3.4 months, respectively. The initial pattern of organ involvement was a good predictor of the nature of disease manifestations at relapse. No relapses were observed in patients whose MPO-ANCA disappeared and remained negative (negative predictive value = 100%).

**Conclusion:**

Regardless of AAV subtype, an increase in MPO-ANCA levels was associated with clinical deterioration. This study suggests that monitoring MPO-ANCA levels in AAV patients in remission could help clinicians to tailor therapy more effectively.

## Introduction

Anti-proteinase-3 (PR3-ANCA) and anti-myeloperoxidase (MPO-ANCA) antibodies are anti-neutrophil cytoplasmic antibodies (ANCA) which are useful markers for diagnosing ANCA-associated vasculitis (AAV) (1–4). According to the Chapel Hill classification, AAVs are necrotizing vasculitides that affect small blood vessels and include three distinct forms: granulomatosis with polyangiitis (GPA), microscopic polyangiitis (MPA), and eosinophilic granulomatosis with polyangiitis (EGPA) (5). MPO-ANCA are present in 70% of patients with MPA, 40% with EGPA, and 10% with GPA (6–8). Patients with EGPA who are seropositive for MPO-ANCA are more likely to experience vasculitic complications (such as renal involvement) and to experience long-term relapses (9). Conversely, cardiac or gastrointestinal complications are more prevalent among seronegative patients (10, 11). However, these antibodies are not entirely specific for AAV as they can also be found in other pathological conditions, such as chronic inflammatory bowel disease or endocarditis, and in healthy individuals (12).

Once an AAV patient has achieved remission with an induction regimen, maintenance therapy involving immunosuppressive drugs such as azathioprine or B-cell-depleting antibodies is required to prevent early relapse (13). Current recommendations regarding the duration and intensity of maintenance treatment do not take into account the individual patient’s risk of relapse. Given the inherent heterogeneity of disease course, identifying factors associated with relapse after remission would benefit patients by decreasing the treatment burden for those at low risk and increasing surveillance for early therapeutic intervention to prevent organ damage in those at high risk of relapse.

There is strong evidence to support the positive and negative predictive value of serial PR3-ANCA measurement in predicting relapses for GPA and MPA patients (14, 15). However, data regarding the utility of serial MPO-ANCA testing in AAV, particularly in EGPA patients, is scarce and inconclusive (16).

This study aims to analyze the predictive value of MPO-ANCA monitoring in AAV patients in remission, with respect to relapse occurrence.

## Materials and methods

### Patient selection and data collection

Patients were identified for this study through the University Hospital Laboratory of Brussels (LHUB-ULB) database, searching for positive MPO-ANCA analyses from January 1, 2014 to January 1, 2024. Medical records were reviewed, and only patients with AAV according to EULAR-ACR^2022^ criteria were included (17). Data pertaining to epidemiological and disease characteristics was collected at baseline (defined as the initial visit) and longitudinally, with follow-up out-patient visits conducted every six months (with a ±1-month window) by an internist, nephrologist, or rheumatologist, per standard of care in our center. Organ involvement was recorded at time of diagnosis and at time of relapse, and was distinguished from treatment-related adverse events (18); the nature of organ damage/dysfunction considered to be AAV-related is detailed in **Supplementary Table 1**. Renal involvement was defined as biopsy-proven AAV-associated glomerulonephritis at diagnosis. The Birmingham Vasculitis Activity Score version 3 (BVASv3) was calculated retrospectively based on medical notes at diagnosis and at time of relapse (18). According to the EULAR definitions for clinical trials, remission was defined as a BVASv3 of 0 with a prednisone-equivalent dose < 7.5mg/day and relapse was defined as a BVAS ≥1 after having achieved remission (19). Relapses were classified according to severity. Major relapses were defined as those involving ≥1 major or vital organ and/or a life-threatening condition, requiring re-initiation of an induction regimen. Minor relapses were defined as those not meeting the criteria for major relapse, requiring modest treatment intensification (increasing glucocorticoid dosage, addition of a second immunosuppressive agent), or continuation of maintenance therapy (20). This study was approved by the Hopital Universitaire de Bruxelles ethics committees, in accordance with the principles of the Declaration of Helsinki.

### ANCA-related laboratory analysis and interpretation

MPO-ANCA detection and quantification was performed using a chemiluminescence enzyme immunoassay (CLEIA) with a positivity threshold at ≥ 20 U/mL (3). All results obtained throughout the follow-up period were recorded. The following patterns were observed with respect to evolution of MPO-ANCA over time: persistence, durable disappearance, and reappearance after a period of negative testing. Among patients with MPO-ANCA persistence, two trends were observed: increasing values (defined herein as ≥ twofold compared to baseline MPO-ANCA level), and decreasing values. For the purpose of this study, two groups were distinguished based on changes in MPO-ANCA over time after remission achievement: the “MPO-ANCA^DECR^” group included patients with disappearance and those with persistent but decreasing patterns, while “MPO-ANCA^INCR^” included patients experiencing either reappearance or a persistent and increasing pattern. Some patients did not undergo serial MPO-ANCA assays during follow-up. In this case, the MPO-ANCA rise was detected at the time of symptom reappearance.

### Statistical analysis

Shapiro-Wilk test was used to assess normality. Continuous variables were presented as mean (standard deviation, SD) or median (interquartile range, IQR) values. Correlations between two continuous variables were investigated by Spearman coefficient. Univariate and multivariate (if appropriate) COX regressions were used to investigate the effect of variables on relapse. Fisher’s exact test was used to assess variation of proportions between groups with binary variables. Survival analysis was realized using Kaplan–Meier survival curves and log- rank tests.

## Results

### Study population

A total of 478 patients tested positive for MPO-ANCA, among whom 73 patients (15.2%) were identified with ANCA-associated vasculitis and underwent regular follow-up; 29 with MPA, 22 EGPA, and 22 GPA. Because EGPA and GPA/MPA patients are now treated differently, these two groups are studied separately in the next sections (13). The remaining MPO-ANCA-positive subjects either had other diseases such as inflammatory bowel disease or idiopathic pulmonary fibrosis (without vasculitis), or had no evidence for an underlying inflammatory condition.

At diagnosis, EGPA patients had a median age of 53 years with a male-to-female ratio of 1.4 (**Table 1**). Median MPO-ANCA level was 60 U/mL and eosinophil count 1950/mm³. Median follow-up was 10 years. For GPA/MPA patients, median age at onset was 57 years with a male-to-female ratio of 0.7. Median MPO-ANCA level was 40 U/mL, and median follow-up was 7 years. Regular follow-up visits at fixed intervals (every 6 ± 1 months) were maintained for all patients up to 24 months. After this period, the follow-up interval was progressively extended in 43 out of 73 patients, with a median follow-up interval of 11.8 months up to month 48 (IQR 9-14).

**Table 1:** Baseline clinical characteristics of EGPA and GPA/MPA patients.

Characteristic	EGPA (n=22)	GPA/MPA (n=51)
Age — yr	53 [44.75–61.5]	57 [45–71]
Male-to-female ratio	1.4	0.7
MPO-ANCA level — U/mL	60 [11–208]	40 [12–134]
Eosinophil count — per mm³	1950 [1693–2400]	—
C-reactive protein — mg/L	120 [80–282]	119 [78–204]
BVASv3 score	10.5 [8.75–21.25]	13 [9–17]
Follow-up — yr	10 [4–11.5]	7 [5–11]

Values are medians and interquartile ranges (IQR). EGPA, eosinophilic granulomatosis with polyangiitis; GPA, granulomatosis with polyangiitis; MPA, microscopic polyangiitis; MPO-ANCA myeloperoxidase antineutrophil cytoplasmic antibodies; BVASv3, Birmingham Vasculitis Activity Score version 3.

**Figure 1:**
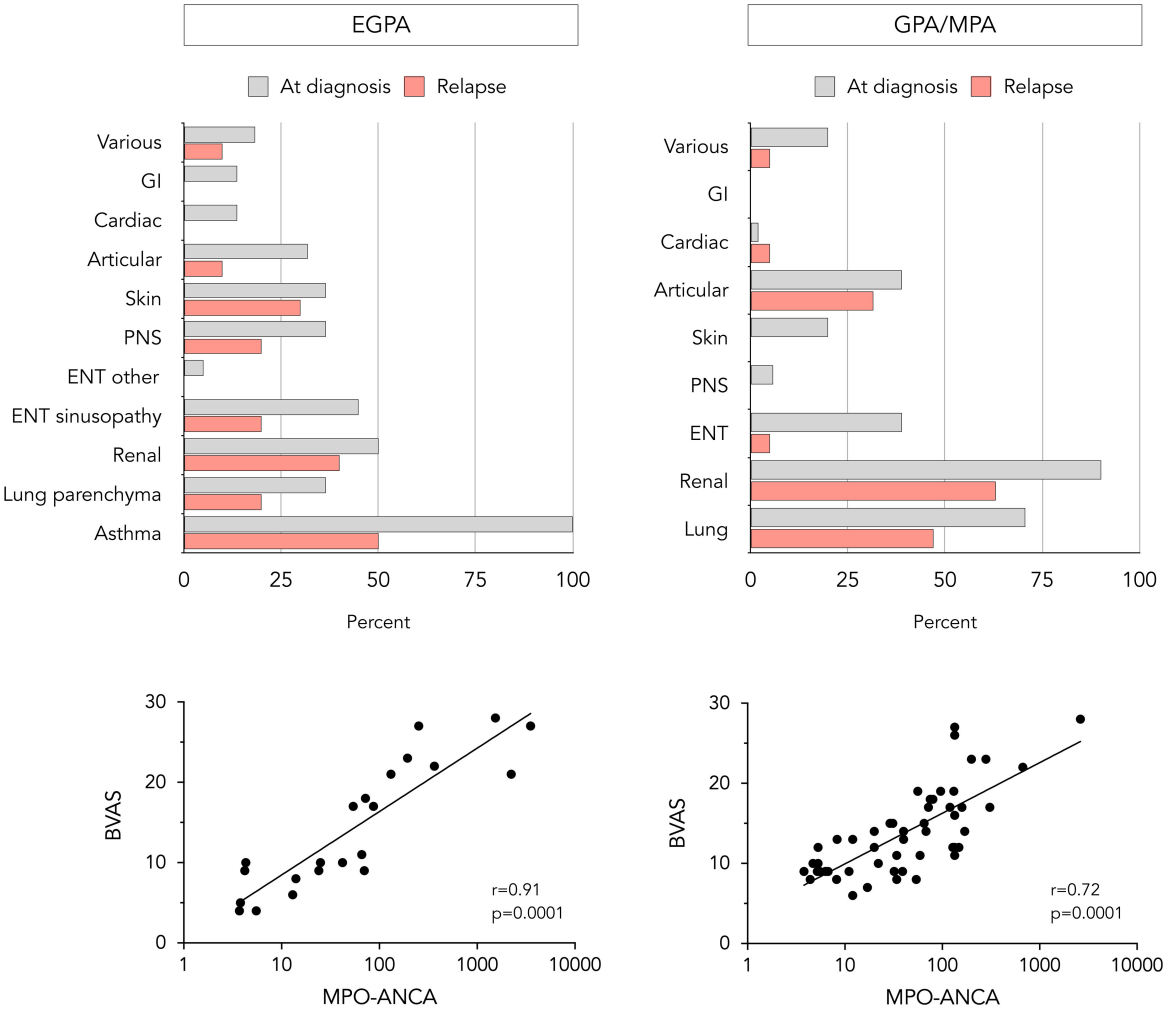
Clinical and laboratory features at diagnosis and relapse in EGPA and GPA/MPA patients. Proportion of EGPA (upper left) and GPA/MPA (upper right) patients presenting with different clinical manifestations at diagnosis and relapse, and correlation between baseline MPO-ANCA level and BVASv3 in EGPA (lower left) and GPA/MPA patients (lower right). The statistical correlation is assessed using Spearman’s test and is expressed as p-value. BVAS, Birmingham vasculitis activity score. ENT, ear-nose-throat; GI, gastrointestinal; MPO-ANCA, anti-myeloperoxidase antibodies; PNS, peripheral nervous system.

Clinical manifestations at diagnosis are shown in **Figure 1**. Pulmonary involvement was the most prevalent feature in EGPA patients; asthma was present in all patients with lung involvement (100%) followed by parenchymal infiltrates (36.4%). Half of the patients exhibited renal and sino-nasal involvement with chronic rhinosinusitis as predominant feature (90.9% of ear-nose-throat [ENT] manifestations). Other systems were less affected at diagnosis. In GPA/MPA patients, renal and pulmonary involvement were observed in over two-thirds of the cases, followed by sino-nasal and articular involvement. In both groups, the majority of patients had a multi-systemic presentation rather than single-organ involvement. A significant correlation between MPO-ANCA level and BVAS at diagnosis was seen in both disease groups (**Figure 1**).

### Characterization of relapses in patients with EGPA and GPA/MPA and impact of baseline disease characteristics and treatment

Among the 22 EGPA patients achieving remission (median time after induction regimen 8 months, IQR 5-11), 10 experienced relapses after a median time of maintenance treatment of 21 months (IQR 15-28). Pulmonary involvement was the most frequently observed manifestation at relapse, namely asthma exacerbations and parenchymal infiltrates in 50% and 20% of relapses respectively (**Figure 1**). Other commonly reported target organs at relapse were kidneys (50% of relapses were proven by repeat biopsy; remaining cases were based on a decline in renal function biomarkers, after excluding other causes of renal deterioration), skin, and ENT which exclusively presented as recurrent rhinosinusitis. Subgroup analysis showed that 30% of relapses manifested as isolated exacerbations of asthma or chronic rhinosinusitis without other systemic involvement, 40% as asthma or rhinosinusitis associated with additional systemic manifestations, and 30% as systemic manifestations without concurrent asthma or sinonasal exacerbation. Regarding relapse severity, two were classified as major, while the remaining were considered minor. Among patients who initially presented with pulmonary and renal involvement, the same organ systems were affected at relapse in 67% and 50%, respectively. Similarly, patients with less common manifestations at diagnosis, such as ocular or central nervous system involvement, appeared to have a higher risk of relapse involving these atypical features.

Of the 51 GPA/MPA patients achieving remission (median time after induction regimen 5 months, IQR 3-7), 19 experienced a relapse after a median duration of maintenance therapy of 23 months (IQR 16-30). In more than half of relapses, renal (75% repeat biopsy-proven) and pulmonary involvement were the leading features (**Figure 1**). Some patients presented articular (31.5%), sino-nasal (5%), or cardiac involvement (5%). The severity of relapse was considered major in 11 out of 19 patients. Similar to EGPA patients, initial pulmonary and renal involvement was associated with a higher risk of the same organ systems being affected at relapse, with recurrence rates of 50% and 53%, respectively.

**Figure 2:**
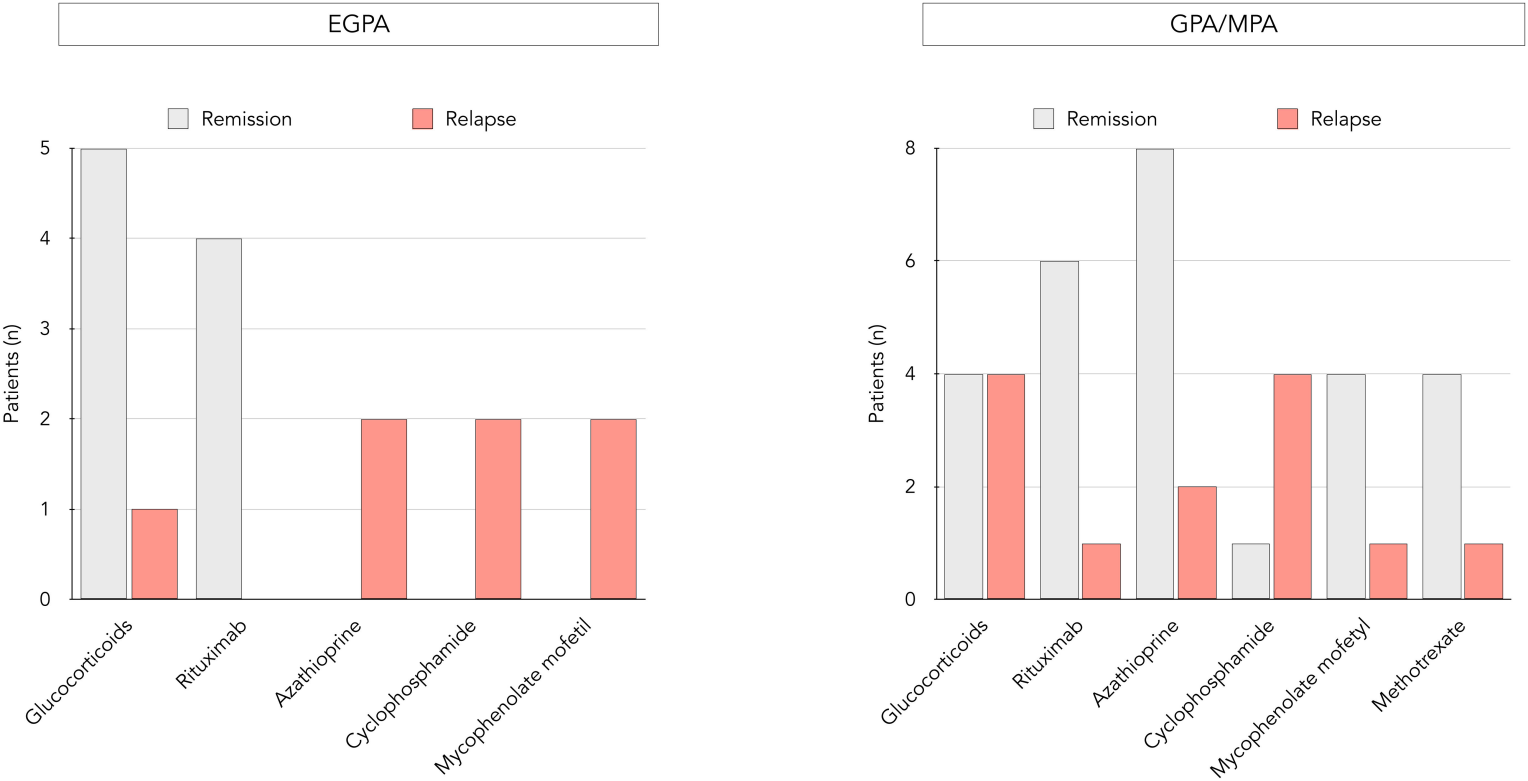
Distribution of maintenance regimens and occurrence of relapses in EGPA and GPA/MPA patients. Distribution of maintenance treatments within the EGPA group (left side) and the GPA/MPA group (right side), each further divided into a remission and a relapse group.

The impact of several variables present at diagnosis on subsequent occurrence of relapses was assessed (**Table 2**). Age, MPO-ANCA, CRP levels and organ involvement were compared in patients experiencing prolonged remission versus relapse, and none was independently associated with relapse at 60 months. In contrast, the nature of maintenance treatment appeared to have an impact on disease course. Indeed, in patients with EGPA, glucocorticoids and rituximab dominated in those with prolonged remission, while use of other immunosuppressants was only seen in those who relapsed (**Figure 2**). In GPA/MPA patients, while maintenance treatment with azathioprine and rituximab was clearly associated with prolonged remission, relapse was seen more often in those receiving cyclophosphamide.

**Table 2:** Evaluation of baseline features as predictive factors for relapse in EGPA and GPA/MPA patients.

EGPA			
Variable	Remission n = 12	Relapse n = 10	P value
Age (yr)	53.0 (46.8–65.0)	53.0 (44.8–59.5)	0.8670
MPO-ANCA (U/mL)	45.0 (11.9–189.5)	48.0 (21.3–113.8)	0.6641
Eosinophil count at diagnosis (/mm³)	1256 (1138-1374)	998 (880-1116)	0.3600
Max eosinophil count (/mm³)	2073 (1637–2241)	1755 (1692–2065)	0.1657
CRP (mg/L)	30.0 (15.0–145.0)	18.5 (14.8–39.5)	0.9066
Organ involvement at diagnosis			
Skin	5	3	0.3860
Gastrointestinal	1	2	0.5214
Renal	4	8	0.0732
ENT	6	6	0.6555
Pulmonary	11	9	0.7112
PNS	5	3	0.4375
Cardiac	2	2	–
Articular	5	2	0.3460
Various	5	2	0.4231

GPA/MPA			
Variable	Remission n = 32	Relapse n = 19	P value
Age (yr)	56.0 (44.3–68.8)	57.0 (51.5–70.5)	0.3901
MPO-ANCA (U/mL)	36.5 (11.8–104.8)	72.0 (21.5–134.0)	0.7634
CRP (mg/L)	22.0 (14.0–45.0)	18.0 (15.0–33.5)	0.5267
Organ involvement at diagnosis			
Skin	7	3	0.3412
Gastrointestinal	0	0	–
Renal	27	19	0.5901
ENT	14	6	0.1287
Pulmonary	20	16	0.4075
PNS	3	0	0.2179
Cardiac	1	0	–
Articular	12	8	0.6432
Various	8	2	0.3817

Patients were divided into a remission and a relapse group, allowing for the comparison of various variables at diagnosis and their influence on relapse. Values are medians and interquartile ranges (IQR). Follow-up period was comparable between groups: 9.8 yr (remission) vs. 10.1 yr (relapse) in EGPA, and 7.3 yr (remission) vs. 6.6 yr (relapse) in GPA/MPA. Cox regression was used to assess statistical difference between groups (p-value).

CRP: C-reactive protein, EGPA: eosinophilic granulomatosis with polyangiitis; ENT: ear nose and throat; GPA: granulomatosis with polyangiitis; MPA: microscopic polyangiitis; MPO-ANCA myeloperoxidase antineutrophil cytoplasmic antibodies; PNS peripheral nervous system.

### Longitudinal assessment of MPO-ANCA status and occurrence of relapses in EGPA

**Figure 3:**
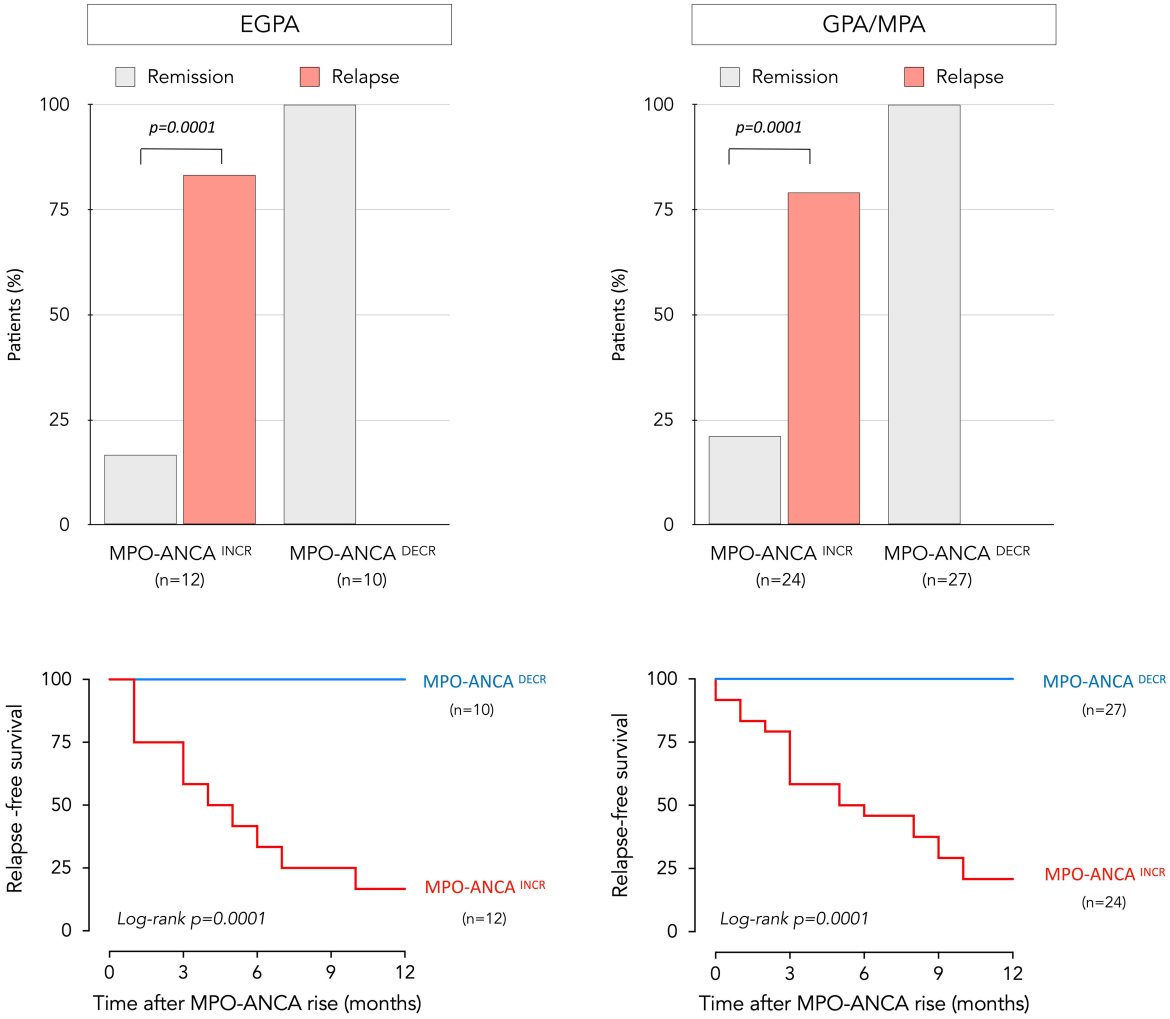
Relationship between relapse and evolution of MPO-ANCA over time in EGPA and GPA/MPA patients. Distribution of patients who remained in prolonged remission and who relapsed, as a function of longitudinal changes in MPO-ANCA levels for EGPA (upper left) and GPA/MPA patients (upper right). The p-value was calculated with Fischer’s test. Relapse-free survival over a 12-month period following an increase in MPO-ANCA levels is represented by a Kaplan-Meier curve in EGPA (lower left) and GPA/MPA group (lower right); no relapses were seen in patients who experienced a decrease in MPO-ANCA levels. Patients experiencing increasing MPO-ANCA after achieving remission are represented in red, while those experiencing decreasing MPO-ANCA are represented in blue. EGPA, eosinophilic granulomatosis with polyangiitis; MPO-ANCA, anti-myeloperoxidase antibodies.

Systematic serial MPO-ANCA monitoring had been conducted, with the exception of 4 EGPA patients in whom testing for ANCA was prompted by a clinical suspicion of relapse. Patients were sub-divided in two groups according to trends in evolution of MPO-ANCA after achieving remission. Twelve experienced increasing MPO-ANCA (“MPO-ANCA^INCR^” group) and 10 were in the “MPO-ANCA^DECR^” group (**Figure 3**). Among the former, 5 had become seronegative during follow-up with subsequent MPO-ANCA reappearance, showing a median time for seroconversion of 4.6 months. A significant association between a rise in MPO-ANCA and relapse was seen (p = 0.0001). All 10 patients who relapsed were in the MPO-ANCA^INCR^ group, with a mean interval between MPO-ANCA rise (reappearance or at least twofold increase) and clinical relapse of 3.6 (+/- 2.1) months. The positive predictive value for relapse following an MPO-ANCA^INCR^ was 83%, with an associated positive likelihood ratio of 6. Of the 10 MPO-ANCA^INCR^ patients who experienced a clinical relapse, 7 developed systemic manifestations (with or without concurrent asthma or rhinosinusitis), while 3 exhibited isolated asthma and/or rhinosinusitis without systemic involvement. The median dose of glucocorticoids used for re-induction of the 10 relapses was 36.75 mg/day. In subgroup analysis based on patients’ recorded body weights, the weight-adjusted dose was 0.5 mg/kg/day in 8 patients with minor relapse and 1 mg/kg/day in 2 patients with major relapse. In contrast, the majority (10/12) of patients with persistent remission did not experience an increase in MPO-ANCA, and no relapses were seen in the MPO-ANCA^DECR^ group.

### Longitudinal assessment of MPO-ANCA status and occurrence of relapses in GPA/MPA

Within this disease subgroup, MPO-ANCA levels had not been monitored regularly and testing was prompted by a clinical suspicion of relapse in 10 patients; otherwise, serial measurements had been performed. Of the 51 GPA/MPA patients, 24 and 27 were classified as MPO-ANCA^INCR^ and MPO-ANCA^DECR^, respectively. Among the 24 MPO-ANCA^INCR^ patients; 13 had become seronegative during monitoring, followed by reappearance of MPO-ANCA with a median time for seroconversion of 5.2 months. During follow-up, 19 patients experienced relapses, preceded by MPO-ANCA reappearance or increase in all cases (**Figure 3**). A significant association between a rise in MPO-ANCA and relapse was seen in these AAV as well (p = 0.0001). The mean interval between MPO-ANCA rise and clinical relapse was 4.6 (+/-3.4) months. The positive predictive value of clinical relapse in MPO-ANCA^INCR^ subjects was 79%, with a likelihood ratio of 6.4. Of the 32 patients with persistent remission, 27 did not experience an increase in MPO-ANCA, and no relapses were seen in the MPO-ANCA^DECR^ group.

### Treatment adjustment and outcomes according to changes in MPO-ANCA levels

**Figure 4:**
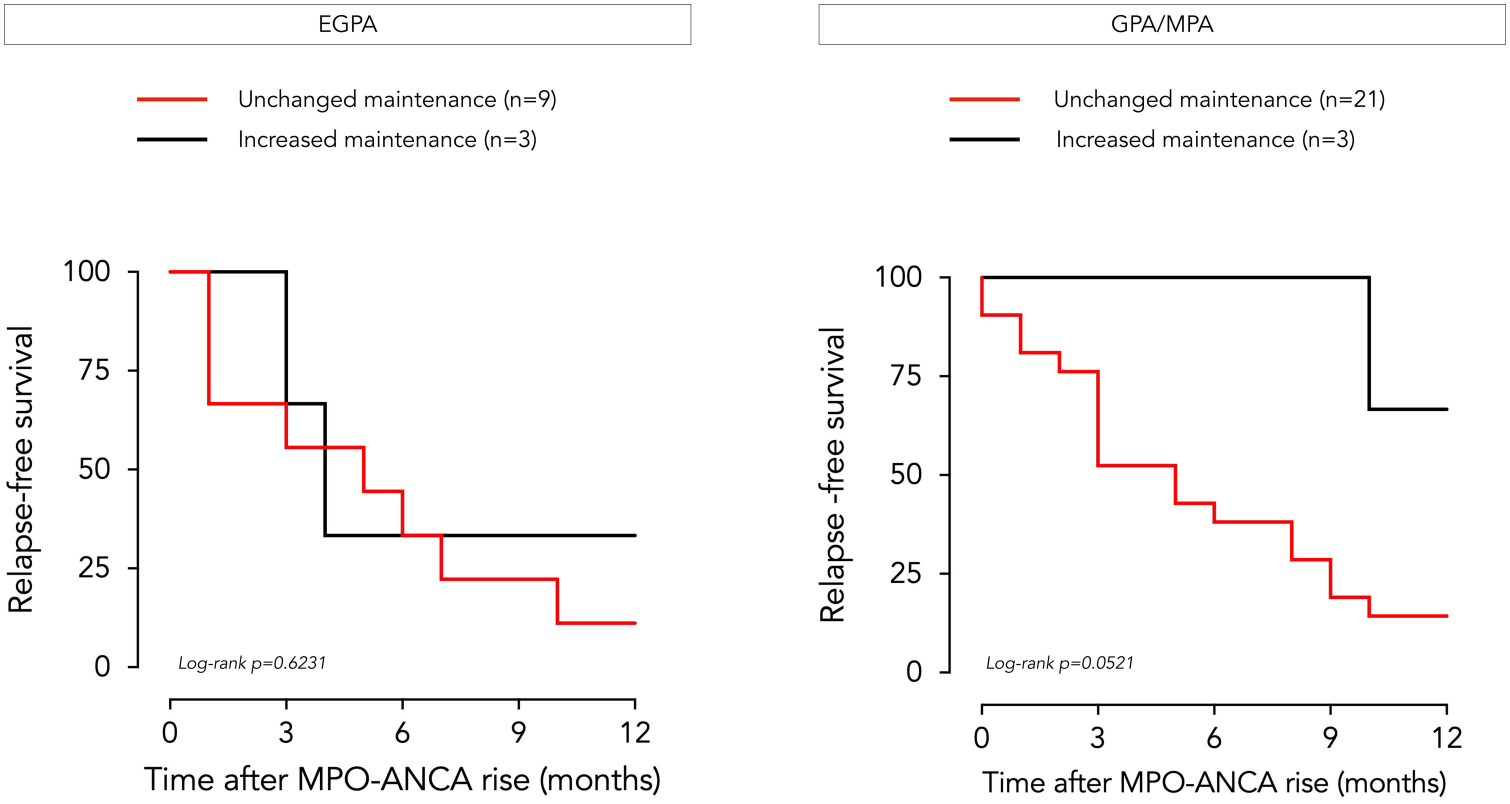
Clinical outcome of EGPA and GPA/MPA patients following MPO-ANCA rise according to treatment adjustment. Relapse-free survival over a 12-month period following an increase in MPO-ANCA levels is represented by a Kaplan-Meier curve for patients with EGPA (left side) and GPA/MPA (right side), according to whether preemptive treatment adjustment was implemented by the physician (in black) or not (in red). EGPA, eosinophilic granulomatosis with polyangiitis GPA, granulomatosis with polyangiitis; MPA, microscopic polyangiitis; MPO-ANCA, anti-myeloperoxidase antibodies.

Of the 36 patients with AAV who experienced an increase in MPO-ANCA levels over time, only 6 (3 with EGPA, 3 with GPA/MPA) received an intensified maintenance regimen before clinical or biological signs of relapse. Glucocorticoid dosing was increased in 4 patients (2 EGPA, 2 GPA/MPA), and immunosuppressive therapy was added in one patient in each disease group (azathioprine for EGPA, methotrexate for GPA/MPA). The likelihood of relapse according to whether treatment was adjusted or not is shown in **Figure 4**. The two EGPA patients who relapsed despite therapeutic escalation experienced asthmatic and sino-nasal exacerbations. The number of patients in whom treatment was intensified following the observed rise in MPO-ANCA was too small to draw any conclusions on the clinical impact of these measures.

## Discussion

This study investigates the value of MPO-ANCA status at baseline and follow-up as a predictive marker for clinical relapse in ANCA-associated vasculitis. Although we categorized patients into two different groups based on the type of vasculitis, our observations led us to similar conclusions for EGPA and GPA/MPA patients. In both groups, baseline MPO-ANCA levels correlated with disease severity at diagnosis (as assessed by BVAS) but were not associated with a higher risk of relapse during follow-up. Therefore, lower MPO-ANCA levels at diagnosis should not reassure the clinician with respect to the likelihood of relapse.

In the present study, no predictive factors for a subsequent relapse were identified at diagnosis in either disease sub-group. However, predictive tools for relapse have already been developed in larger cohorts. Samson et al. developed the « French Vasculitis Study Group Relapse Score » (FRS), applied at diagnosis to assess the risk of relapse for GPA/MPA (21). Their criteria include PR3-ANCA positivity, age ≤75 years and glomerular filtration rate ≥30 mL/min/1.73m^2^. In addition, McClure et al. developed a model to predict the risk of relapse at the time of the last rituximab infusion for GPA/MPA patients, including age >60 years, male gender, ANCA positivity, prior relapses, ENT involvement, prednisone dose, and concomitant immunosuppressive therapy (22). This score can be used to stratify patients into high and low relapse-risk groups, thus assisting clinicians in decision-making during follow-up.

In EGPA, although eosinophils are known to contribute to pathogenesis, eosinophil levels at diagnosis did not predict an increased risk of relapse during follow-up. It should be noted however that the blood eosinophil count may be only slightly elevated at diagnosis, firstly it is hypothesized that at the late vasculitic phase these cells migrate to extravascular tissues (23) and secondly because patients often already receive glucocorticoid therapy before referral (24). We therefore also traced previous eosinophil counts, but found no relationship between maximal eosinophil counts and likelihood of relapse either (25).

By excluding MPO-ANCA seronegative EGPA, several clinical observations in this series were biased. First, more vasculitic manifestations such as renal, cutaneous, or neurological involvement were observed. In contrast, cardiac or gastrointestinal involvement are more common in the eosinophil-dominant seronegative form. Second, the relapse rate (45%) was higher than that observed in the literature, which generally combines both types of EGPA (30-35% relapse rate) (23, 26, 27). The higher relapse risk conferred by MPO-ANCA seropositivity is still unclear. It could be explained by the dual role played by MPO-ANCA, both marker and effector in the pathophysiology of the disease (28). Their mere presence isn’t directly pathogenic and requires several inflammatory stimuli (C5a, IL-8, TNF-α) for neutrophils to express myeloperoxidase on their surface, which can then be targeted leading to vascular inflammation. Their exact pathophysiological role remains unclear and is still debated. In a genome-wide association study, Lyons et al. reported that MPO-ANCA–positive EGPA shares specific clinical features and an HLA-DQ association with other MPO-AAV, in which the pathogenic role of ANCA is well established (29). These findings support the hypothesis of a dual-pathogenesis model in EGPA, whereby eosinophilic inflammation drives asthma, chronic rhinosinusitis, and eosinophil-mediated organ damage, while MPO-ANCA mediates vasculitic manifestations. In line with this, recent studies have reported vasculitic relapses occurring upon rising MPO-ANCA titers despite normal eosinophil counts in patients treated with anti-IL-5 therapies, providing clinical support for the pathogenic role of MPO-ANCA in the vasculitic component of the disease (30). In our cohort, 70% of relapses following an MPO-ANCA rise presented with systemic vasculitic manifestations, consistent with this model. Additionally, 70% of these relapses also involved asthma or sino-nasal exacerbations, highlighting the challenge of localized disease control despite absence of peripheral eosinophilia.

In GPA/MPA patients, we observed a predominance of renal involvement, as previously reported in association with MPO-ANCA. In contrast, ENT involvement was less common in this sub-group, also consistent with previous observations in presence of MPO-ANCA (31). The relapse rate was lower compared to EGPA, which can be explained by the higher risk of relapse for seropositive EGPA patients (23, 26, 27). Furthermore, within the other two forms, GPA is associated with a higher relapse rate compared to MPA (32). Combining both in the same group therefore reduces the average relapse rate. However, GPA and MPA remain chronic conditions with a risk of relapse and require appropriate follow-up.

Our results support the predictive value of MPO-ANCA dynamics once remission has been achieved, and confirm their position as a valuable monitoring tool during follow-up. Indeed, we show that an increase in MPO-ANCA levels is associated with a risk of clinical deterioration, and conversely, persistent negativity indicates a very low (potentially absent) risk of relapse (negative predictive value of 100% in our cohort). Regardless of the AAV subtype, the initial pattern of organ involvement appears to be a good predictor of the nature of disease manifestations at relapse. Moreover, it is important to keep in mind that relapses in EGPA can manifest in two distinct forms: a “localized” phenotype, limited to asthma and sinonasal symptoms, and a “systemic” phenotype, characterized by vasculitic and eosinophilic features. Although clinically different, both forms of relapse reflect uncontrolled disease activity and therefore require therapeutic escalation.

The nature of the molecule chosen for maintenance treatment may have an impact on the risk of relapse. Patients with EGPA receiving glucocorticoids alone or rituximab had a reduced risk of relapse compared to those treated with other agents in our study (33). This could be explained by the phenotypic distinction of relapses observed in EGPA (airways-limited versus systemic) which highlights the two active inflammatory axis in EGPA, and therefore the possibility of relapse of one pathway despite effective control of the other (e.g., asthmatic relapse despite vasculitis control). Glucocorticoids provide effective control both by depleting eosinophils and by suppressing the systemic vasculitic component. The efficacy of rituximab on the eosinophilic component remains unclear, given its consistent association with glucocorticoids during induction therapy. On the other hand, the reduced risk of relapse in our ANCA-positive EGPA patients receiving rituximab could be rather applied to vasculitic relapses and explained by the efficacy of rituximab on ANCA-vasculitic inflammation. Other immunosuppressants used in our cohort likely do not provide satisfactory eosinophilic control, as illustrated by the marked percentage of relapses presenting as asthma and/or rhinosinusitis exacerbations (70% of relapses). It should be noted that no patient was treated with eosinophil-depleting biologics which have recently been approved in many countries for this indication. Indeed, the MIRRA trial reported the efficacy of mepolizumab (an anti-IL-5 antibody) in reducing relapse rates and it is now recommended as a suitable alternative to rituximab for patients with non life- or organ-threatening disease (34). More recently the MANDARA trial established that benralizumab (an anti-IL-5R antibody) is non inferior to mepolizumab in terms of remission and relapse rates (35). Furthermore, it is worth noting that the percentage of ANCA-positive patients included in both trials was low. In GPA/MPA patients, the risk of relapse appeared to be reduced with rituximab or azathioprine, in line with the EULAR-ACR^2022^ recommendations (13). Guillevin et al. compared both treatments and concluded that rituximab was superior to azathioprine, making it the first choice for maintenance therapy (36). The MAINRITSAN II trial subsequently confirmed the importance of rituximab maintenance in reducing relapse rates (37). A recent study demonstrated that relapse rates were lower in patients whose rituximab reinfusions were guided by B cell repopulation compared to those guided by a rise in ANCA (38). Presumably, enhanced efficacy of the former approach may be related to interference with recurrence of ANCA formation, given their potential pathogenic contribution to disease activity. This highlights the importance of combined biological monitoring, assessing not only B cell-associated biomarkers but also those reflecting other inflammatory pathways, namely neutrophils, such as neutrophil extracellular traps (NETs) which have recently shown promise in this respect (39).

**Figure 5:**
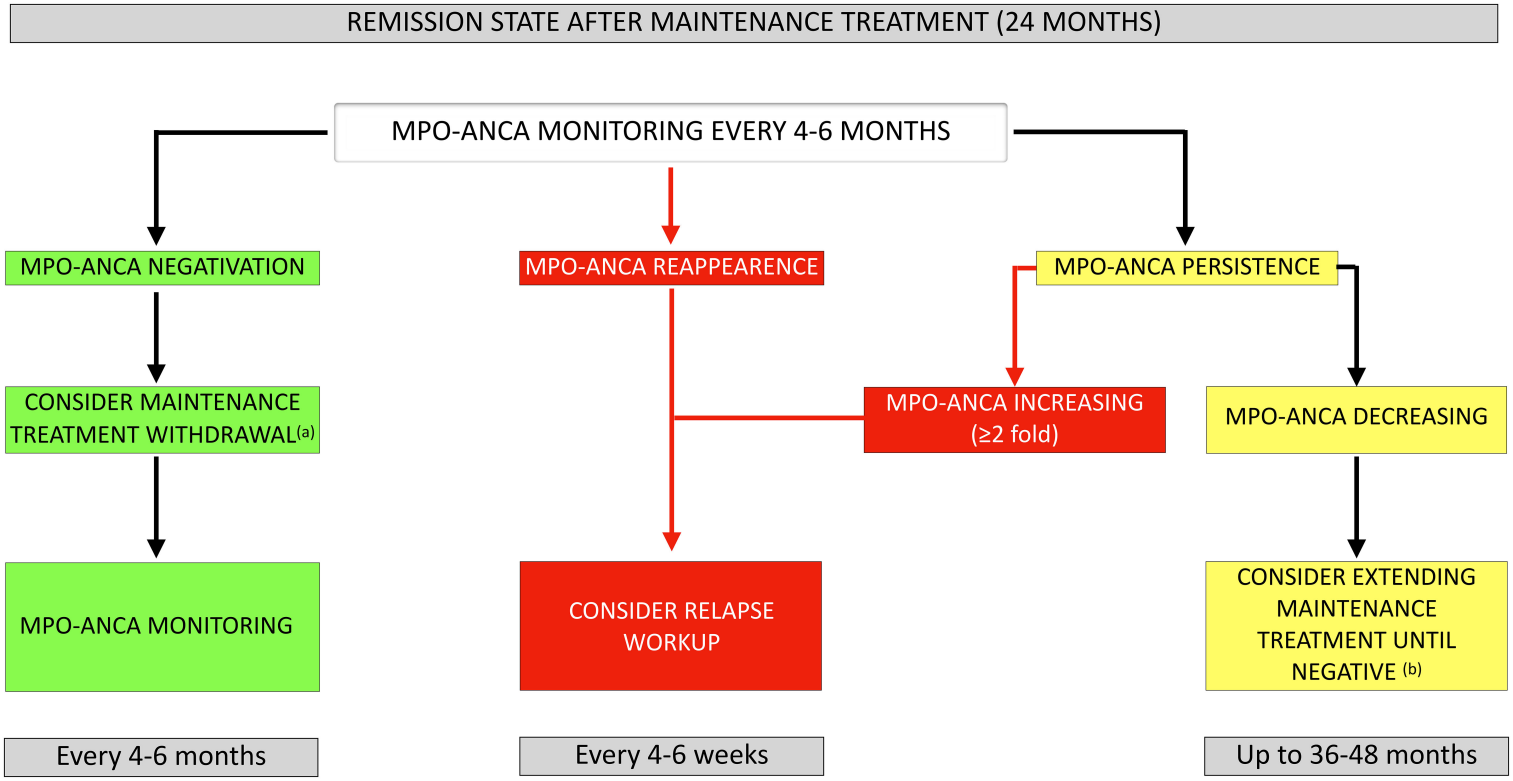
Proposed approach for MPO-ANCA monitoring in AAV patients once remission has been achieved. **(a)** In patients receiving rituximab as maintenance therapy, the relapse risk prediction model developed by McClure et al. can be used to evaluate whether extension of rituximab maintenance is warranted: consider rituximab withdrawal in low-risk vs. extending rituximab up to 36 months in high-risk patients. **(b)** Consider maintenance therapy extension up to 36–48 months until negative seroconversion based on individual relapse risk-benefit balance and potential long-term toxicity of immunosuppressive agents. MPO-ANCA, anti-myeloperoxidase antibodies.

On the basis of our findings, we propose here a common approach for anti-MPO-seropositive EGPA and GPA/MPA patients in remission using MPO-ANCA monitoring (**Figure 5**). A similar strategy has already been proposed by Casal Moura et al. for GPA and MPA, but our study confirms that it can also be applied to EGPA (40). We suggest MPO-ANCA monitoring every 4–6 months, based on the mean interval (4.6 and 5.2 months for EGPA and GPA/MPA, respectively) observed for MPO-ANCA reappearance (once they have become undetectable) herein. After completion of the recommended 24-month period of maintenance treatment (based on EULAR^2022^ recommendations), follow-up could be adapted according to MPO-ANCA status during follow-up. In patients who remain MPO-ANCA negative, discontinuation of maintenance therapy could be considered followed by appropriate MPO-ANCA monitoring. The reappearance of MPO-ANCA after a period of negativity should raise suspicion of disease reactivation, since we have shown a significant association with relapse. Similarly, patients with persistently elevated MPO-ANCA levels, showing a progressive increase, should be considered at risk of relapse. The average time between MPO-ANCA increase and relapse was 3.6 months and 4.6 months for EGPA and GPA/MPA, respectively. A comprehensive workup should be performed to look for clinical or biological evidence of relapse every 4–6 weeks (in order to prevent serious and/or irreversible damage by timely intensification of treatment). For the specific case of persistently elevated MPO-ANCA levels with progressive decline, maintenance therapy may be extended up to 36–48 months until negative seroconversion, based on individual relapse-risk (increased by persistent MPO-ANCA positivity), potential adverse effects of prolonged immunosuppression, co-morbidities, and patient preference. This approach is proposed based on the results from the pooled analysis of the MAINRITSAN trials, as well as EULAR^2022^ recommendations (13, 41).

This study highlights a trend among clinicians not to preemptively escalate immunosuppression in the face of an isolated rise of MPO-ANCA titers without clinical manifestations. This approach is well-justified and in line with current recommendations (13). However, our results suggest that the vast majority of patients who experience an MPO-ANCA rise without receiving treatment escalation will relapse within 6–12 months. Yamaguchi et al. investigated a preemptive therapeutic approach when ANCA levels increase and demonstrated a significant reduction in associated relapse risk (4.5% with preemptive approach vs. 82.9% without) (42). Although the sample size was insufficient to demonstrate a statistically significant difference in our cohort, preemptive therapeutic escalation appeared more effective in GPA/MPA compared to EGPA. This may be explained by the nature of relapse manifestations: renal involvement in GPA/MPA patients, typically responsive to immunosuppressive agents, versus asthmatic/sinonasal features in EGPA, sometimes refractory to immunosuppressants, requiring optimization of topical treatment and/or sinus surgery and/or addition of mepolizumab/benralizumab (10).

This study has several limitations, related in great part to its retrospective nature. We assessed clinical relapses by calculating the BVAS retrospectively, based on medical records. Some patients were not newly diagnosed, and because some patients were referred from other centers, data on induction regimens and prior relapses was not always available or complete. MPO-ANCA levels were not systematically assessed at strict intervals and it is likely that the interval between measurements was influenced by the level of suspected disease activity. Some patients consulted between planned visits because of appearance of signs of relapse, which distorts the interval between MPO-ANCA rise and relapse observed. Maintenance therapy varied widely among patients, as management guidelines have evolved between 2014 and 2024. The relatively small size of the study cohort limits the power of statistical analyses. Future prospective randomized multicenter studies are therefore needed to support our findings.

In conclusion, this retrospective study evaluating the predictive value of MPO-ANCA at baseline and over time for relapse in AAV shows that increases in ANCA titers are significantly associated with clinical deterioration, irrespective of the AAV subtype. Although limited by its design, our study lays the foundations for future prospective multicenter studies, with the ultimate goal of improving patient outcomes by reducing unnecessary exposure to prolonged immunosuppressive treatment and adjusting maintenance regimens as appropriate.

## Data Availability

The raw data supporting the conclusions of this article will be made available by the authors, without undue reservation.
