# Supplementary material for: Clinical value at baseline and follow-up of myeloperoxidase-antibodies in ANCA-associated vasculitis
Source: Front Immunol. 2025 Sep 1;16:1649708. doi: 10.3389/fimmu.2025.1649708 (PMC12434123; doi:10.3389/fimmu.2025.1649708)
Supplement: Supplementary file 1 [file Table1.docx]

# Supplementary material

**Table 1**. Clinical manifestations sorted by systems according to BVASv3.

| Systems involved | Clinical manifestations |
| --- | --- |
| *ENT involvement* | *Bloody or mucopurulent discharge with sinusopathy, nasal polyposis, nasal septum damage, granulomas, stridor due to subglottic stenosis, conductive hearing loss, sensorineural hearing loss* |
| *Lung involvement* | *Late-onset asthma, dyspnea due to parenchymal damage (alveolar hemorrhage, nodules, infiltrates) or endobronchial involvement, respiratory failure requiring ventilation support* |
| *Renal involvement* | *Proteinuria > 0.2g/24h, haematuria > 10 RBC/uL, serum creatinine > 125 umol/L or > 30% rise of baseline creatinine, > 25% fall in creatinine clearance* |
| *Skin involvement* | *Purpura, ulcers, gangrene, subcutaneous nodules, erythema nodosum* |
| *Articular involvement* | *arthritis* |
| *PNS involvement* | *Objective sensory deficit, single or multiple motor nerve palsies due to mononeuritis multiplex* |
| *Heart involvement* | *Myocarditis, pericarditis, cardiomyopathy with heart failure* |
| *Gastrointestinal involvement* | *Gastrointestinal bleeding, peritonitis, bowel ischemia* |
| *Various* | *Hypothalamo-hypophyseal infiltration, aseptic meningitis, scleritis,episcleritis, uveitis,* |

Other etiologies were excluded before considering the condition as a complication of AAV.
